# Protein Kinase A Detection in Human Urine Samples

**DOI:** 10.3390/jcm10184096

**Published:** 2021-09-10

**Authors:** Angela Ragone, Alessia Salzillo, Annamaria Spina, Silvia Zappavigna, Michele Caraglia, Luigi Sapio, Silvio Naviglio

**Affiliations:** Department of Precision Medicine, University of Campania “Luigi Vanvitelli”, Via L. De Crecchio 7, 80138 Naples, Italy; angela.ragone@unicampania.it (A.R.); alessia.salzillo@unicampania.it (A.S.); annamaria.spina@unicampania.it (A.S.); silvia.zappavigna@unicampania.it (S.Z.); michele.caraglia@unicampania.it (M.C.); silvio.naviglio@unicampania.it (S.N.)

**Keywords:** protein kinase A, biomarker, serum, urine, ELISA, Western blotting

## Abstract

Actively involved in tumor maintenance, cAMP-dependent protein kinase A (PKA) has been proposed as a putative biomarker in cancer. Recently, an active PKA form has been identified in human sera and PKA autoantibodies have been detected in cancer patients. However, their serum functions, as well as diagnostic significance, remain largely unknown. Although several PKA detection assays have been developed, none refer to a laboratory diagnostic procedure. Among these, ELISA and Western blotting (WB) assays have been employed in PKA detection. Since, to the best of our knowledge, there are no data showing its presence in human urine samples, herein, we explore the possibility of PKA’s existence in this biological specimen. Interestingly, among the 30 screened urines by quantitative sandwich ELISA, we recognized detectable PKA levels in 5 different samples, and of those two exhibited a considerable high concentration. To corroborate these results, we also evaluated PKA’s presence in both positive and negative ELISA urines by WB. Remarkably, immunoblotting analysis confirmed PKA’s existence in certain, but not in all, human urine specimens. Despite being quite preliminary, these findings firstly identify PKA in urine samples and provide evidence for its potential clinic usage as a diagnostic analyte in laboratory medicine.

## 1. Introduction

Adenosine 3′,5′-cyclic monophosphate (cyclic AMP, cAMP) is considered one of the leading second messengers in mammalian cells [1]. As the primary intracellular target for cAMP, protein kinase A (PKA) is dynamically involved in crucial functions, such as cell metabolism, proliferation, and differentiation [2]. Two distinct isoforms, named PKA-I and PKA-II, have been recognized in humans to date, which differ in regulatory (R) subunits, cAMP binding affinity, and cellular localization [3]. In this respect, PKA type I usually has a higher affinity for cAMP and cytoplasmic localization, whereas type II is anchored to subcellular structures and compartments [4]. As inactive heterotetramer, PKA holoenzyme takes shape when two cAMP molecules bind each of the regulatory subunits, causing the release and activation of the catalytic domain. Besides cAMP stimulation, PKA can also be activated independently by nuclear factor κB (NF-κB), transforming growth factor-beta (TGF-β), and SMAD family members [5,6]. Sharing core motifs with all other kinases, catalytic (C) subunits phosphorylate serine and threonine residues on specific substrates in both the cytosol and nucleus, of which cAMP-response element binding protein (CREB) represents the main target for PKA [7]. Although five distinct presumed DNA segments have been identified, almost all entire C-subunits in human cells result from PRKACA and PRKACB genes, which encode for Cα and Cβ, respectively [8].

The different numbers of R and C entities, as well as their tight regulation, further denote how proper PKA operation is critical for cellular homeostasis. In this regard, dysregulation in either expression or activation is usually associated with the onset of different pathological conditions, including neurological diseases, endocrine disorders, and cancers [9,10,11,12]. In malignancies, for instance, depending on the spatial distribution and activation time, PKA can exert both proliferative and inhibiting effects [13,14]. To note, differential PKA-I/PKA-II expression usually results in opposite clinical outcomes; in fact, whilst PKA-I is predominantly expressed in tumor cells and is generally associated with more serious clinic-pathological features, PKA-II constitutes the most representative isoenzyme in growth-arrest appearance [13]. Despite the existence of conflicting findings, PKA’s involvement in cancer is further corroborated by the dynamic role of its downstream targets, such as CREB [15]. 

Recently, an extracellular PKA form (ECPKA) was recognized first on the surface of LS-174T human colon carcinoma cells, and later in human sera [16,17]. Biochemically and immunologically related to the intracellular species, ECPKA phosphorylates the synthetic peptide substrate Kemptide, while the employment of PKI (Walsh–Krebs inhibitor) entirely blocks its enzymatic activity [18]. Contrary to the inside portion, ECPKA activation seems unrelated to cAMP and the inactive form is completely missing. However, mutual influences between intra and extracellular PKA have been described. In this respect, vector-mediated Cα or RIα overexpression upregulates both intracellular PKA-I and ECPKA in cancer cells, whereas RIIβ genetic induction increases PKA-II and downregulates ECPKA [19]. Although its biological role remains largely unknown, ECPKA from HCT-116 and LnCAP cancer cell media has been reported to inhibit angiogenesis in in utero chicken embryo chorioallantoic membrane assay [20]. Interestingly, ECPKA activity has been found to be significantly higher in sera from tumor patients than in cancer-free ones, and ECPKA autoantibodies have also been detected in malignancies [17,18,21]. In this respect, Wu and colleagues have recently recognized significant differences in PKA catalytic Cα (PKA-Cα) levels between volunteers and gastric cancers, but not colorectal, in sera [22]. Analogous cancer-related findings have also been obtained in canine species [23,24]. 

Considering the emerging significance as a potential biomarker, several PKA detection assays have been developed and tested over the years, including ELISA-based assay, WB, and, latterly, time-gated luminescence and fluorescence assays [25,26,27,28]. Nevertheless, none refer to a laboratory diagnostic procedure and PKA has not yet been accepted in as a clinical diagnostic parameter. Whilst these procedures are enabled to recognize PKA in serum, plasma, and marginally in follicular fluids, its detection in other body fluids currently remains undefined [29]. With this in mind, the present study was conceived to address the chance of identifying PKA in human urine samples, the most-used biological fluid in diagnostics together with blood. Employing both ELISA and WB as detection techniques, we estimated the possibility of measuring PKA, and more specifically the catalytic subunit α (PKA-Cα), in a restricted number of urine specimens.

## 2. Materials and Methods

### 2.1. Human Biological Samples

Blood specimens were collected from 14 volunteers, including 5 men and 9 women, aged between 3 and 76 years (mean 47.1 ± 23.8). Serum separation was achieved through centrifugation at 3000× *g* for 20 min. Unpaired urine samples were obtained from 30 different individuals divided into 15 men and 15 women in the age group of 1 to 78 years (mean 35.7 ± 27.0). Clinical and pathological information was neglected during samples’ recruitment and PKA assessment. No statistically significant differences were detected in gender within the serum and urine group. After collection, aliquots of serum samples were analyzed either within two hours by WB or stored at −20 °C and evaluated in 2–6 months by ELISA. Urine samples were directly kept in the freezer until analysis instead. All specimens were provided following the provision of informed patient consent and approved by the Ethics Committee of University of Campania (ID no. 618 approved on 7 September 2018).

### 2.2. Cell Culture 

Normal mouse embryonic fibroblasts (NHI 3T3) and human lung carcinoma cells (A549) were obtained by American Type Culture Collection (Manassas, VA, USA). The employed cell lines were grown in Dulbecco’s Modified Eagle’s Medium (DMEM) (ECM0728L, Euroclone, Pero, Italy) containing 10% FBS (ECS0180L, Euroclone) and 100 mg/mL penicillin-streptomycin (ECB3001D, Euroclone), and kept in a controlled temperature and atmosphere (37 °C and 5% CO_2_). 

### 2.3. Cell Protein Extraction

Cell pellet was lysed in 3–5 volumes of RIPA buffer (R0278, Sigma-Aldrich, St. Louis, MO, USA) supplemented with protease (#5871, Cell Signaling Technology, Danvers, MA, USA) and phosphatase inhibitors (P2850, Sigma-Aldrich). Following 30 min of incubation on ice, cell lysate was spun down (14,000× *g* for 15 min at 4 °C) and the supernatant was recovered. Finally, the protein concentration of each sample was defined by the Bradford method (39222, Serva, Heidelberg, Germany). 

### 2.4. Serum and Urine Protein Concentration

Biuret reagent was employed to define the total protein amount of human biological samples. For additional information on system and assay technology, refer to the ARCHITECT System Operations Manual, Section 3 (Abbott, Chicago, IL, USA). 

### 2.5. Samples Preparation

Laemmli sample buffer 2X (S3401, Sigma-Aldrich) or 6X (home-made) was added to every sample before boiling at 95 °C for 6 min.

### 2.6. Western Blotting

Approximately 10–30 µg of cell protein extract and 5–50 µg of serum or urine proteins were loaded in 10% acrylamide gels. Later, proteins were transferred onto a nitrocellulose membrane (GEH10600008, Cytiva, Marlborough, MA, USA) by Mini Trans-Blot (Bio-Rad Laboratories, Hercules, CA, USA). In order to reduce both noise and aspecific bond, potential free spots on nitrocellulose film were covered with non-fat milk (5% *w*/*v*) (A0830, PanReac Applichem, Chicago, IL, USA) for one hour. Anti-PKA-Cα (sc-903, Santa Cruz Biotechnology, Dallas, TX, USA) was subsequently incubated overnight at 4 degrees. The next day, HRP-conjugated goat anti-rabbit (#7074, Cell Signaling Technology) was added to the membranes for one hour at room temperature. Washing with TBS Tween-20 (TC287, HIMEDIA, Mumbai, India) preceded and followed each single procedure. Lastly, using enhanced chemiluminescence reagent (E-IR-R301, Elabscience, Houston, TX, USA), immunoblotting bands were detected and acquired by Chemi-Doc XRS (Bio-Rad Laboratories). 

### 2.7. PKA ELISA Assay

The Human PKA ELISA kit was performed in agreement with the manufacturer’s procedure (MBS034086, MyBiosource Inc., San Diego, CA, USA). Specifically, 50 µL of serum and/or urine sample were added to each 48-microwell coated with mouse monoclonal antibody immunogen against recombinant full-length human PKA. Subsequently, 100 µL of HRP-conjugated rabbit polyclonal antibody, reactive towards a recombinant fragment of human PKA-Cα, were mixed in each well for one hour at 37 °C. After this time, wells were washed four-fold and incubated with 50 µL of chromogen solution A and B for 15 min, respectively. Using 50 µL of stopping solution, optical density (O.D.) was recognized at 450 nm by a microplate reader (iMark, Bio-Rad Laboratories). Standard samples were employed to set the calibration curve (ranging from 31.2 to 1000 U/L) and to establish the relative serum and urine PKA concentration.

### 2.8. Statistical Analysis

The average value ± standard deviation of biological and/or technical replicates was reported. Differences in the mean between the blank and samples were calculated using Welch’s t-test analysis. P values of less than 0.05 were recognized as significant. Image J 1.52 software analysis (NIH, Bethesda, MD, USA) was employed for WB quantification, whereas GraphPad Prism 8 (GraphPad Software Inc., San Diego, CA, USA) was used to interpolate unknown PKA concentrations from a linear standard curve.

## 3. Results

### 3.1. Human Urine Samples Can Sporadically Contain Detectable PKA Levels

To investigate whether PKA could be detected in urine samples, we randomly recruited 30 volunteers and screened them for the analyte of interest by quantitative sandwich ELISA. 

**Figure 1:**
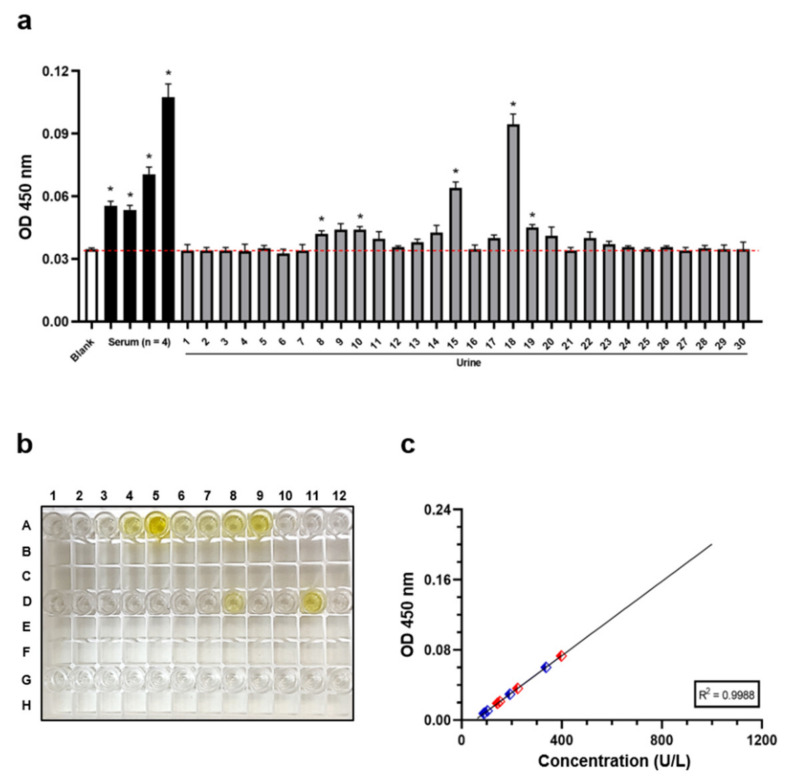
Evaluation of PKA’s presence in human urines. (**a**) Four serum and thirty urine samples were tested with the purpose of estimating PKA’s presence by ELISA assay. O.D. value ± Standard Deviation (SD) of each individual specimen is reported in graph. (**b**) Representative ELISA Strip-Wells. A1 Blank; A2-A5 Standards; A6-A9 Serum; A10-A12, D1-D12, and G1-G12 Urine. (**c**) PKA standard curve and relative levels in positive biological samples. Red dots indicate the PKA concentration in serum, blue dots indicate it in urine instead. * *p* < 0.05 by Welch’s *t*-test.

Since this methodological approach is currently employed in determining the PKA levels in blood samples, we also included four different sera in our own analysis [22,30]. As expected, four out of four sera exhibited O.D. values higher than blank noise, thus suggesting the presence of fluctuating PKA levels (Figure 1). Surprisingly, checking the absorbance scales within the examined urine specimens, we detected a high O.D. rate in two distinct samples, namely U15 and U18 (Figure 1a and 1b). Nevertheless, 5 out of 30 samples displayed statistically significant higher values compared with the background signal. 

The subsequent usage of both the standard concentration gradients and curve fitting software defined the standard curve and the relative analyte amount (Figure 1c). Whilst the PKA concentration ranged between 88.9 and 336.8 U/L within the positive urines, a similar but slightly higher extent was detected in sera (from 143.2 to 398.2 U/L). 

Altogether, the obtained ELISA results suggest the possible presence of PKA in the urine samples. 

### 3.2. Western Blotting Analysis Supports the Presence of PKA in Human Urine Samples

Among the available detection assays, WB has been proposed as an alternative method for revealing PKA’s presence in biological samples, in human sera [18,27]. 

**Figure 2:**
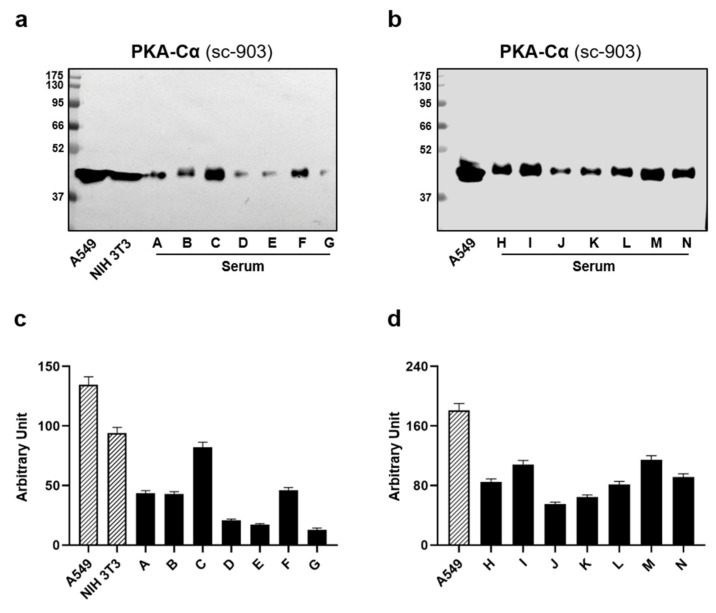
Estimation of PKA-Cα levels in human sera. In total, 50 µg of human serum were analyzed by WB assay for PKA-Cα levels. In total, 30 µg of A549 and NIH 3T3 whole cell extracts were employed as the antibody positive control instead. (**a**) Representative WB film including A549, NIH 3T3, and serum samples A–G. (**b**) Representative WB of A549 and sera H-N. (**c**) Densitometric analysis relating to panel a. (**d**) Densitometric analysis of panel b.

To corroborate this assumption, we evaluated the PKA-Cα levels in 14 different human sera by immunoblotting analysis. Notably, Figure 2 shows a unique and distinct band in every tested serum, with an analogue molecular weight compared to the PKA catalytic subunit α from cell extracts, which we used as an internal positive control. 

With the purpose of sustaining the presence of PKA even in urine, we applied WB analysis to a selected number of both positive (U15, U18, and U19) and negative (U2, U3, and U30) ELISA urine samples. Moreover, we also included NIH 3T3 lysate as one of the recommended whole cell extracts for antibody control.

**Figure 3:**
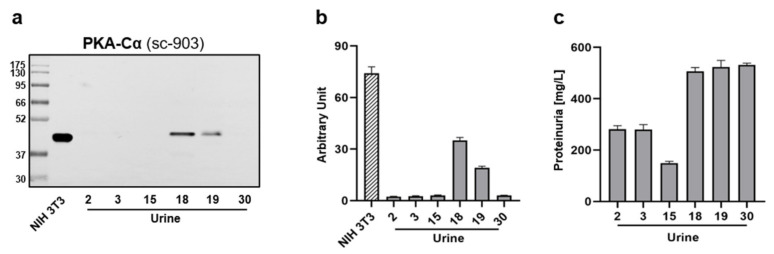
Assessment of PKA-Cα levels in human urines. In total, 45 µL of U2-, U3-, and U30-negative and U15-, U18-, and U19-positive ELISA urine, as well as 10 µg of NIH 3T3 were examined for PKA-Cα levels by WB. (**a**) Representative WB image. (**b**) Densitometric analysis of panel a. (**c**) Protein amount within WB loaded urine samples. Data are shown as average rate ± SD.

Interestingly, besides the NIH 3T3-positive test, Figure 3a displays a band appearance in correspondence with U18 and U19, whereas no signal, as expected, was revealed in reaction to U2-, U3-, and U30-negative ELISA samples. Strangely, despite U15 displaying a considerable O.D. in the immunosorbent assay, no PKA signal was observed in WB (Figure 3a,b). Looking at the protein content, U15 presented a very low amount compared with the other WB employed samples (Figure 3c). Considering that the maximum volume was loaded in each well, we presumed that the volume/protein ratio might represent a potential relevant technical limitation in WB-mediated PKA detection. 

Taken together, these findings confirm the presence of PKA in some human urine samples and further recognized WB as a potential alternative tool for detecting this analyte in different body fluids.

## 4. Discussion

Early detection and diagnosis represent the first successful strategy to defeat cancer. As such, the UK Office for National Statistics has recently confirmed how a much higher survival rate is conditional on an early detection stage compared with a late or advanced grade [31]. Therefore, detecting neoplastic diseases before clinical symptoms’ appearance could improve survival and decrease morbidity. 

In this scenario, blood-related markers have provided an additional tool for early cancer recognition. Different biochemical parameters have been approved in the diagnosis of malignancies over the years, including carcinoembryonic antigen (CEA), cancer antigen (CA) CA19-9, CA15-3, CA125, and prostate-specific antigen (PSA) [32]. Nevertheless, a lack of sensitivity, as well as specificity, of these markers has severely limited their employment in cancer screening, and thus novel tumor biomarkers are still required to reveal in advance this terrible illness [33,34,35]. 

The existence of extracellular PKA in human peripheral blood has resulted in speculation on its usage as a potential tumor-related biomarker. However, even though initial findings have supported this hypothesis, the clinical significance of ECPKA has never taken off. This inconsistency is probably the result of different factors, which include the low number of available studies and the undefined extracellular PKA physiological function [36,37,38]. Moreover, the lack of standardization in the laboratory procedure has further prevented the PKA from entering in clinical practice.

Here, we provided supplementary evidence supporting WB analysis as a surrogate method for PKA detection in human specimens. Currently, only two distinct studies employ WB as a practicable approach to detect PKA in biological samples [18,27]. However, it should be kept in mind that whilst Mani and coworkers claimed that ECPKA expression has been evaluated by WB, without showing any data [27], Cho and collaborators performed PKA-Cα immunoprecipitation before applying WB [18]. Therefore, as far as we know, there is no obvious evidence regarding the use of this technique in PKA detection. Our results first display WB’s ability in detecting PKA in biological samples, even though several technical and procedural limitations should be taken into account for clinical use, including the lack of validation and standardization. Although the above weaknesses could be minimized in the future, others might persist, such as the influence of the volume/protein ratio in signal detection. In our setting, for instance, the U15-positive ELISA sample turned out to be negative in WB, likely as a consequence of the low protein amount. Indeed, although the maximum volume was applied for all samples, the loaded content only reached 5 µg in U15, unlike the others, whose values were approximately 10 µg or higher. On the contrary, although U30 had the highest protein concentration among the WB screened samples, no signal was revealed. The inability to detect PKA in U15 sample by WB could also be the result of the different analytical basics by which these two assays recognize PKA. Moreover, since neither in ELISA nor in WB the exact binding locations have been mapped, we cannot rule out that different PKA portions were detected. In light of the obtained results, it is more believable to conceive WB as a potential confirming rather than screening test, as is currently the practice in HIV diagnosis [39].

Beyond the technical issues, the achieved results convincingly indicate the presence of PKA in certain, but not in all, human urine specimens. In this respect, two distinct vulnerabilities arise from our study, namely the limited number of processed samples, and the loss of an association between PKA appearance and specific clinic-pathological features. With this in mind, we do know that much still needs to be done in overcoming the mentioned limitations and increasing its clinical relevance. Therefore, our future studies and investigation will be dedicated to improving these aspects and to better defining the clinical significance of this promising novel analyte. In this respect, identifying potential PKA substrates in both serum and urine, as well as improving its biochemical characterization, might help to outline PKA’s usefulness in diagnostics. Specifically, we will look into the possibility of detecting phosphorylated PKA, as well as phosphorylated downstream targets, in these samples for future directions.

## 5. Conclusions

Illustrating for the first time the possibility of detecting PKA in the very common and largely used biological sample of urine, our findings further support its potential clinic usage and could open up new horizons in laboratory medicine.

## Data Availability

The data presented in this study are available on request from the corresponding author.
